# Visiting Out-of-Home Places When Living With Dementia: A Cross-Sectional Observational Study: Visiter des lieux hors du domicile lorsque l'on vit avec une démence: étude transversale observationnelle

**DOI:** 10.1177/00084174211000595

**Published:** 2021-03-22

**Authors:** Isabel Margot-Cattin, Catherine Ludwig, Nicolas Kühne, Gunilla Eriksson, André Berchtold, Louise Nygard, Anders Kottorp

**Keywords:** ACT-OUT, Community, Dementia, Occupational gaps, Social participation, ACT-OUT, communauté, démence, écarts occupationnels, participation sociale

## Abstract

**Background.:**

Persons living with dementia face a reduction of their life space outside home and disengagement from participation, linked to places visited.

**Purpose.:**

This study explored stability and change in perceived participation in places visited outside home and its relationship with occupational gaps among older adults.

**Method.:**

Older adults living with (*n* = 35) or without (*n* = 35) dementia were interviewed using the Participation in ACTivities and Places OUTside Home (ACT-OUT) questionnaire and the Occupational Gaps Questionnaire (OGQ). Data analysis used descriptive and inferential statistics.

**Findings.:**

The group of people living with dementia reported significantly fewer places (*p* < .001) visited than the comparison group and having abandoned more places visited (*p* < .001) than the comparison group. The number of occupational gaps was significantly different between groups (*p* < .001).

**Implications.:**

Participation outside home is not influenced in a uniform and straightforward way for persons living with dementia; the shrinking world effect appears differently in relation to types of places.

## Abstract (fr)

**Contexte de recherche.:**

Les personnes vivant avec une démence font face à une réduction de l'espace extérieur et se désengagent de la participation sociale et des lieux visités à l'extérieur.

**Objectif de la recherche.:**

Cette étude vise à explorer la stabilité et les changements dans les lieux visités à l'extérieur du domicile et leurs relations avec les écarts occupationnels auprès de personnes âgées.

**Méthodologie.:**

Des personnes âgées vivant avec (n=35) ou sans démence (n=35) ont été interviewées avec les questionnaires “Participation in ACTivities and Places OUTside Home (ACT-OUT) et “Occupational Gaps Questionnaire”. L'analyse des données a été menée à l'aide de statistiques des criptives et inférentielles.

**Résultats.:**

Le groupe de personnes âgées vivant avec une démence rapporte visiter significativement moins (*p* < .001) de lieux que le groupe de comparaison et abandonne également plus de lieux (*p* < .001). Le nombre d'écarts occupationnels était aussi significativement différent entre les deux groupes (*p* < .001).

**Implications.:**

La participation à l'extérieur du domicile n'est pas influencée de manière uniforme et directe pour les personnes vivant avec une démence; l'effet de diminution du monde extérieur se produit de manière différenciée selon les types de lieux visités.

## Introduction

Outside home participation is an important part of everyday life, as well as a challenge, for community-dwelling older adults living with early-to-moderate-stage dementia. Although an age-related reduction in activities outside home is well documented (Baker et al., 2003), there is further evidence demonstrating a substantial “shrinking world” for persons living with dementia (Duggan et al., 2008). Recent research has shown that little by little they disengage from social activities outside home, like being a member of associations or clubs, and going to social gatherings, expositions, or concerts (Argyle et al., 2017; Kuosa et al., 2014). They also face driving cessation, which increases their risk of disengagement (Marottoli et al., 2000).

Notwithstanding, persons living with dementia strive to maintain their preferred patterns of engagement in participation outside home (Brorsson et al., 2011). In order to maintain participation outside home, they need to navigate and reach places that support their engagement in everyday occupations. Spaces become places when persons repeatedly engage and perform occupations there (Meijering et al., 2019; Townsend et al., 2009). In this study, we consider visiting places as an essential attribute of participation outside home. Although not all spaces become places for all individuals, it supports a subjective perspective on participation (Brown et al., 2004). As persons living with dementia engage in occupations in diverse places, they might create experiences of performance and perceive their participation outside home (Chaudhury et al., 2020) that they are then able to tell about. Places hold individual meanings, embracing social, cultural, and political aspects, as well as spatial and temporal dimensions (Hand et al., 2017). Thus, the “shrinking world” effect not only outlines a diminishing of a physical out-of-home space, but also a progressive loss of the occupations embedded in those places.

In addition, the decline in participation among persons living with dementia is complex and nuanced: many places show commonalities in participation rather than significant differences between groups of persons living with and without dementia (Chaudhury et al., 2020; Gaber et al., 2019). Yet, to better understand the “shrinking world” effect, it is important to identify places in which participation changes with the course of the disease. Although the “shrinking world” effect assumes a general linear reduction in out-of-home participation, there might be places that individuals keep visiting overtime, as well as places that show an increase of visits with the course of the disease. Having better insights into both changes and stability is cardinal for further specifying the characteristics of the “shrinking world” assumed in dementia and for better understanding their need for support.

Persons living with dementia and their families report various explanations for reduction in participation. First, they report challenges in finding one’s way and getting to intended places without taking too much time, energy, or worry, and ultimately without getting lost (Sheehan et al., 2006). A decreasing use of public transportation and driving also seems to account for a reduction in participation (Scott et al., 2019). Further, visiting spaces and/or places outside home is linked to increased risks of falling, of experiencing embarrassing situations, and of having traffic accidents, for those who keep driving, and difficulties as pedestrians (Hunt et al., 2010). Older adults with dementia worry about situations encountered during activities outside home that are problematic, like paying in the store (Brorsson et al., 2013). Such experiences interfere with opportunities to participate. Whenever individuals become unable to access or use outside spaces and/or places, they may feel “disconnected” or “sealed out” (Duggan et al., 2008).

Persons living with dementia might not want this disengagement and are not satisfied with it (Low et al., 2018). According to Magasi et al. (2009), being able to engage in desired activities, as well as not to engage in undesired ones, is an important aspect of participation. Losing these opportunities leads to an “occupational gap” (Eriksson & Tham, 2010), which in itself is inversely correlated with satisfaction in participation. A recent study showed that there is a significant association between participation and satisfaction in everyday occupations that one wants to perform (Bergström et al., 2017). Bringing together these various pieces of evidence leads us to assume that persons experiencing a shrinking world effect might also experience a loss of participation opportunities, that is, occupational gaps.

This study aims to explore the differences between persons living with dementia and persons without known cognitive impairments in relation to the perception of places visited outside home, maintenance and abandonment of places, and perceived occupational gaps. Research questions were elaborated as follows: (a) Are there differences between persons living with dementia and persons without known cognitive impairments in relation to reported places visited outside home? (b) Are there differences between persons living with dementia and persons without known cognitive impairments in relation to reported maintenance and abandonment of places outside home? (c) Is there a relationship between perceived occupational gaps and reported places outside home visited among persons living with dementia and persons without known cognitive impairments?

## Methods

This observational exploratory survey-based interview study relies on a case-control cross-sectional design (Groves et al., 2009), based on the comparison of two groups of older adults: one group of persons living with dementia, abbreviated as “g-plwd,” and one group of persons without dementia, called the comparison group, and abbreviated as “g-comp.”

### Data Collection Instruments

The Participation in ACTivities and Places OUTside Home (ACT-OUT) Questionnaire was used to collect data on spaces and/or places and activities in combination that individuals participate in, including changes that have occurred in the past or may occur in the future. We refer to places here, although we are aware that not all items in ACT-OUT would be considered as places by all participants. The ACT-OUT Questionnaire was developed in three languages (English, French, and Swedish) through a cross-cultural study and consists of three parts (I−III) (Margot-Cattin et al., 2019). In the French version used here, Part I includes a list of 25 predetermined types of places. Items are grouped into four domains: A/consumer-administrative-and-self-care-places (*n* = 7); B/places-for-medical-care (*n* = 5); C/social-cultural-spiritual-places (*n* = 6); and D/places-for-recreational-and-physical-activities (*n* = 7). One non-determined place at the end of domain D allows the participants to tell of additional places important for them. Participants were asked three questions for each of the 25 places in Part I: (a) if they visit the place now, (b) if they earlier had visited the place, and (c) if they could see themselves visiting the place in the future. For a place like hairdresser, the interviewers would ask: “Do you go to a hairdresser?” “Did you go to a hairdresser in the past?” “Do you see yourself going to a hairdresser in the future?” Answers are given as yes/no. Part II entails detailed questions with a set of fixed response alternatives about factors potentially influencing participation in places, like activities performed, transportation means, accompanying persons, risk perception, and familiarity. Part III consists of general questions about perceived participation, life satisfaction, and attitudes toward risk-taking and stress factors. In this study, we used data from Part I of the ACT-OUT. An interview using ACT-OUT takes between 40 and 80 minutes to be completed. As of now, there are no psychometric publications for ACT-OUT.

The Occupational Gaps Questionnaire (OGQ) was used to collect data on perceived occupational gaps. It was initially developed for people with Acquired Brain Injury (ABI) to measure participation in everyday occupations (Eriksson et al., 2013). The OGQ is a checklist with 30 items, representing instrumental activities of daily living, work or work-related activities, and leisure and social activities, both inside and outside home. For each activity, the person is requested to answer yes or no to the following questions: (a) Do you perform this activity? (b) Do you want to perform this activity? The internal validity of the scale has been tested in cognitively impaired populations with stroke or ABI, using a Rasch analysis, and was found acceptable (Eriksson et al., 2013). For this study, OGQ was translated from Swedish to French. Two-forth translations (T1, T2), a combined version (T12), then two back translation (T3, T4), and a combined version (T34) were used for consideration by the research team to finalize the end translation (T5) (Beaton et al., 2000; Wild et al., 2005). The French translation of OGQ was then refined and implemented using cognitive interviews (Willis, 2005). Three rounds of three interviews were conducted by three interviewers, interspaced by adaptation processes organized through a dual-panel translation approach (Hagell et al., 2010). The French version was tried out with persons living with dementia before the data collection started, as the OGQ has not earlier been used in this population, nor is it validated for this population. The OGQ takes about 15 minutes to be completed.

The Montreal Cognitive Assessment (MoCA) (Nasreddine et al., 2005) was used as a comprehensive screening tool, covering all key domains of cognition (Lischka et al., 2012), for assessing and describing the level of cognitive functioning for both groups. The MoCA has no ceiling effect and is sensitive to early cognitive deficits (Thomann et al., 2018). We applied a cut-off score of 22 out of 30 for increased specificity while maintaining the sensitivity of the instrument, and for compensating higher age groups (Hsu et al., 2015; Lischka et al., 2012). The MoCA takes approximately 15 minutes to complete.

Finally, a socio-demographic questionnaire was used to describe the sample, covering age, gender, rural/urban location, educational level, type of household, driving, type of retirement pension, and health condition, because these are elements potentially influencing out of home participation.

### Participant Sampling, Recruitment, and Ethical Considerations

Participant sampling occurred in five French-speaking cantons of Switzerland between December 2015 and May 2017. Participants were required to be able to communicate in French and were excluded if they had any mobility disability that significantly impacted their ability to be mobile in the community, like a wheelchair. All were informed that their participation in the study was voluntary and that they were free to leave if need be.

Persons living with dementia (*n* = 35) were recruited through memory clinics, day hospitals, and the Swiss Alzheimer’s association. Diagnosis of dementia was established by medical professionals from memory clinics in the area covered by the study. For the purposes of optimal recruitment, we did not discriminate between the various types of dementia. The competence of persons living with dementia to provide informed consent is a central ethical consideration in research, as the condition of dementia can impair an individual’s capacity to make decisions (Keady et al., 2018). Potential participants were informed by the designated staff member at the memory clinics. Individuals gave verbal assent for permission to be contacted by telephone by our team’s research assistant to explain the study purpose and expectations. Those who agreed to participate in the interview were scheduled for a date, time and location; based on her/his convenience, mostly at their home. On the day of the interview, written informed consent was obtained from the participants and a significant other (typically a family member), as per request of the ethical committee. We also asked for oral assent to participate from the participants and audio recorded it. Furthermore, we implemented a consent monitoring (Dewing, 2007) throughout the data collection to ensure no stress or burden from participating in the project (McKeown et al., 2010). It enables researchers to include consent communicated through behaviour and non-verbal means by the person with dementia. This scheme is recommended when involving persons living with dementia in research (Sugarman et al., 2007). When significant others were present, it was to provide comfort and support in the interview (Nygård, 2006).

Participants (*n* = 35) were recruited through senior associations and ads in grocery stores. The comparison group was aimed to match the group of persons living with dementia, but not individually paired, regarding age, gender, living areas and settings, and education level; thus, recruitment strategies for the comparison group targeted specific regions, age groups, or living areas, for example, to bring the means of the comparison group closer to the other group on those variables.

The sample size was estimated based on the difference on the total number of places visited between the 26 older adults and the 5 persons living with dementia who completed ACT-OUT in the study presenting its development (Margot-Cattin et al., 2019). An ethical authorization (protocol 452/15) was obtained from the “Commission cantonale d’éthique de la recherche sur l’être humain (CER-VD)” in Lausanne, Switzerland.

### Data Collection Procedures

The setting for data collection based on face-to-face interviews was tested beforehand with persons living with dementia in a previous study (Margot-Cattin et al., 2019). We tested the impact of asking the questions in a face-to-face interview and adapted it by rephrasing the question multiple times if needed or taking breaks if the participant showed stress (Dewing, 2007), and by training the interviewers. Participants were able to provide clear answers to the questions asked them.

The same two experienced occupational therapists—including the first author—who collected data in the previous study, conducted all interviews in this study too, using three instruments and questions about demographics in one session which lasted from one hour and fifteen minutes to two hours. Participants preferred not to have a second interview scheduled, although it was offered to them. The way interviews were conducted was harmonized (Pezalla et al., 2012). The interview session was structured by the order of the instruments used (socio-demographic questionnaire, ACT-OUT, OGQ, MoCA). Both interviewers were knowledgeable about various communication challenges in the condition of dementia and how to adopt effective strategies to optimize the responses. The strategies included: (a) appropriate interview setting to ensure confidentiality and comfort, like the home; (b) taking time to build rapport with the person; (c) adapting the pace of the interview; (d) rephrasing the questions if needed; (5) paying attention to lapse in concentration and possible distractions, difficulty in finding words, and possible anxiety; and (6) listening attentively and being empathic (Cridland et al., 2016). It has been shown that persons living with dementia are able to share their perspectives on their own participation outside home (Alzheimer Europe, 2011; Chaudhury et al., 2020).

### Data Analysis

Descriptive statistics were conducted to report participants’ characteristics. Group differences were assessed by means of inferential statistics: *t*-test were used for the continuous outcomes (age and cognitive level (MoCA)), and Fisher’s exact test for the ordinal outcomes (gender, education, income, living arrangement, and health mobility limitations), calculated with IBM SPSS, version 25, to calculate the tests. To reflect the geography of Switzerland, the living areas were categorized into rural (less than 1,000 habitants), peri-urban (between 1,000 and 10,000 habitants), and urban (more than 10,000 habitants) (von der Mühll et al., 2016). To categorize the education level, we used the International Standard Classification of Education (ISCED 2011) (UNESCO Institute for Statistics, 2012), adapted into three levels (1 = primary/secondary school, 2 = apprenticeship, and 3 = university degree). Income was determined based on the “three-pillars” system,^1^ the social security plan for retirement in Switzerland.

According to ACT-OUT, a place is *applicable* when the participant reports going there either in the present, past, or future. An applicable place can be *currently visited*, meaning that the participant goes there in the present; and it can be *affected by change*, meaning, for example, that the participant does not go there in the present, but has gone in the past or will go in the future. A place is *not applicable* if the participant reports neither going there in the present, past, or future.

First, three independent sample *t*-tests were used for the comparison of the overall participation between the two groups in applicable, currently visited, and affected by change places (ACT-OUT), and one *t*-test was used on the total number of occupational gaps (OGQ) (see Table 1). Second, for each place in ACT-OUT, the difference between the groups for currently visited places was calculated with Fisher’s exact test. The number of currently visited places per domain was also summed up, and *t*-test was used to determine whether both groups had the same mean (see Table 2). Third, to evaluate change in currently visited places, the counts from the past participation in each visited place were subtracted from the present participation and expressed as ratios per visited place, and the ratios were also compared between the two groups (see Table 3). A line has been drawn at the ratio of 10% of abandonment for both groups, as that is the ratio found for the number of places affected by change for the comparison group, which could be considered as the “norm ratio” of abandonment for the sample (see Table 1). Fourth, Fisher’s exact test was used for each item in OGQ, of which only the items showing a significant difference are reported.

**Table 1:** Perceived Participation According to ACT-OUT and OGQ

**ACT-OUT and OGQ Variables**	**G-plwd (*n* = 35)**	**G-comp (*n* = 35)**	**CI of Mean Difference**	**Coefficient: *t*-test, Fisher’s Exact Test**	***P*-value**
Description of Participation (ACT-OUT, OGQ)					
No. of applicable places (ACT-OUT, max = 25)				*t* = −.883	.380
Mean (*SD*)	21.66 (2.14)	21.23 (1.91)	−1.39; .53		
No. of currently visited places (ACT-OUT, max = 25)				*t* = 3.893	<.001
Mean (*SD*)	15.83 (3.34)	18.91 (3.28)	1.50; 4.66		
No. of places affected by change (ACT-OUT, max = 25)				*t* = −6.587	<.001
Mean (*SD*)	5.80 (2.78)	2.31 (2.56)	−4.76; −2.20		
Percentage of applicable places affected by change	26.75%	10.88%			
Total no. of gaps (OGQ, max = 30)				*t* = −4.132	<.001
Mean (*SD*)	4.94 (3.80)	1.89 (2.16)	−4.54; −1.57		

**Table 2:** Domains and Places in ACT-OUT (Part 1) That Show a Significant Difference (*p* < .05) in Participation Between the Groups

	**G-plwd**	**G-comp**	**CI (95%) of Mean Difference**	**Coefficient: *t*-test, Fisher’s Exact Test**	***P*-value**
	**(*n* = 35)**	**(*n* = 35)**			
Domain A: Commercial and Administrative Places					
No. of visited places: Domain A				*t* = 4.622	<.001
(max = 7)					
Mean (*SD*)	4.43 (2.11)	6.31 (1.15)	1.07; 2.70		
A1: Grocery store				4.200	.040
Yes	27	33			
No	8	2			
A4: Pharmacy				8.929	.006
Yes	23	33			
No	12	2			
A6: Bank/post office				22.680	<.001
Yes	16	34			
No	19	1			
A7: Administration office				11.283	.002
Yes	12	26			
No	23	9		** **	** **
Domain B: Medical Care Places					
No. of visited places: Domain B				*t* = −2.653	.010
(max = 5)					
Mean (*SD*)	3.26 (0.98)	2.71 (0.71)	−.95; −.13		
B5: Day hospital				32.083	<.001
Yes	22	0			
No	13	35			
Domain C: Social, Spiritual, and Cultural Places					
No. of visited places: Domain C				*t* = 3.144	.002
(max = 6)					
Mean (*SD*)	3.71 (1.25)	4.66 (1.25)	.34; 1.54		
C6: Entertainment and cultural place				10.944	.002
Yes	17	30			
No	18	5			
Domain D: Places for Recreation and Physical Activities					
No. of visited places: Domain D (max = 7)				*t* = 2.661	.010
Mean (*SD*)	4.11 (1.18)	5.03 (1.65)	.23; 1.60		
D4: Cottage or summer house or chalet				7.529	.012
Yes	7	18			
No	28	17			
D7: Transportation centre (train station, airport)				6.629	.019
Yes	19	29			
No	16	6			

**Table 3:** Hierarchies of Counts of Differences and Ratios Between Past and Present Participation in Places Visited Outside Home, Indicating Places Abandoned (Highest Count and Ratio) or Retained (Lowest Count and Ratio) Among the Groups (ACT-OUT)^2^

4 Domains	Type of Place	G-plwd (*n* = 35)				G-comp (*n* = 35)			Type of Place	4 Domains
		Past /Present	Difference	Ratio	Places Abandoned	Ratio	Difference	Past /Present		
C3	Senior centre, social club	24/10 **(−14) 58.33%**			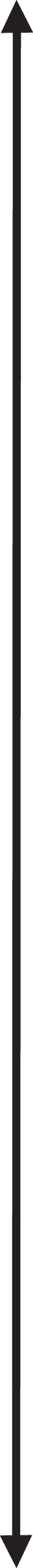	**48.27% (−14)** 29/15			Sports facility	D6
D6	Sports facility	24/10 **(−14) 58.33%**				**38.46% (−5)**13/8			Hospital, health centre	B2
D4	Cottage, summer house	16/7 **(−9) 56.25%**				**27.59% (−8)** 29/21			Building for worship	C4
A6	Bank, post office	34/16 **(−18) 52.94%**				**25.00% (−5)** 20/15			Therapy	B4
C6	Entertainment, cultural places	30/17 **(−13) 43.33%**				**21.74% (−5)** 23/18			Cottage, summer house	D4
A7	Administration office	21/12 **(−9) 42.86%**				**18.18% (−4)** 22/18			Senior centre, social club	C3
B4	Therapy	21/12 **(−9) 42.85%**				**12.12% (−4)** 33/29			Transportation centre	D7
D7	Transportation centre	32/19 **(−13) 40.62%**				**10.34% (−4)** 30/26			Administration office	A7
A4	Pharmacy	33/23 **(−10) 30.30%**				**9.09% (−3)** 33/30			Entertainment, cultural places	C6
A2	Mall, supermarket	33/24 **(−9) 27.27%**				**8.82% (−3)** 34/31			Dentist’s office	B3
A3	Small retail store	34/25 **(−9) 26.47%**				**7.40% (−2)** 27/25			Park, green area	D2
C4	Building for worship	27/20 **(−7) 25.92%**				**6.06% (−2)** 33/31			Mall, supermarket	A2
C5	Cemetery, memorial place	26/20 **(−6) 23.07%**				**6.06% (−2)** 33/31			Small retail store	A3
A1	Small grocery store	35/27 **(−8) 22.86%**				**5.71% (−2)** 35/33			Small grocery store	A1
A5	Hairdresser	35/28 **(−7) 20.00%**				**3.57% (−1)** 28/27			Neighbourhood	D5
D2	Park, green area	23/19 **(−4) 17.93%**				**3.57% (−1)** 28/27			Cemetery, memorial place	C5
D3	Forest, mountain, lake, sea	34/28 **(−6) 17.65%**				**3.12% (−1)** 32/31			Garden in your backyard	D1
B3	Dentist’s office	35/31 **(−4) 11.43%**				**3.03% (−1)** 33/32			Restaurant, cafe, bar	C2
C2	Restaurant, cafe, bar	32/30 **(−2) 6.25%**				**2.94% (−1)** 34/33			Pharmacy	A4
D5	Neighbourhood	33/31 **(−2) 6.06%**				**2.94% (−1)** 34/33			Hairdresser	A5
C1	Friend, family member’s place	35/33 **(−2) 5.71%**				**2.86% (−1)**35/34			Bank, post office	A6
B1	Doctor’s office	35/34 **(−1) 2.86%**				**0% (0)**35/35			Friend, family member’s place	C1
B5	Day care	22/22 **(0) 0%**				**0% (0)** 0/0			Day care	B5
B2	Hospital, health centre	15/15 **(0) 0%**				**0% (0)** 35/35			Doctor’s office	B1
D1	Garden around the house	30/30 **(0) 0%**				**0% (0)** 31/31			Forest, mountain, lake, sea	D3
**Places Retained**										

Finally, the relationship was explored between the total number of currently visited places (ACT-OUT) and the total number of gaps (OGQ), among both groups, using Spearman’s rank correlation coefficient (two-tailed) (Howell et al., 2017). The cut-offs applied to measure the strength of correlation used in this study follow Cohen’s (1988) guidelines from social sciences (.1 to .3 = small association, .3 to .5 = medium association, and .5 to 1.0 = large association).

All analyses were undertaken with a significance threshold set at *p* < .05.

## Findings

The participants (*n* = 70) were living in the community in their own dwellings. The group of persons living with dementia ranged between 65 and 94 years of age, while the comparison group ranged between 67 and 92 years (g-plwd mean (*SD*) = 77.66 (8.35), g-comp mean (*SD*) = 77.86 (7.72)). Majority of them were women (g-plwd = 19 (54%), g-comp = 23 (65%)). Most of the participants had an apprenticeship or a diploma degree (g-plwd = 48%, g-comp = 48%), or completed primary or secondary school (g-plwd = 25%, g-comp = 28%), while a minority graduated from higher education (g-plwd = 25%, g-comp = 22%). Minority of them lived alone (g-plwd = 31%, g-comp = 40%) and very few lived in urban settings (g-plwd = 17%, g-comp = 31%). Health limitations were reported by half of the participants in both groups. The groups are generally well matched (age: *p* = .917, gender: *p* = .333, education: *p* = .950, income: *p* = .380, living arrangement: *p* = .618, setting: *p* = .384, health limitations: *p* = .999), except for the expected significant difference in the cognitive level, reported through the MoCA score (g-plwd mean (*SD*) = 17.74 (5.56), g-comp mean (*SD*) = 26.09 (2.07), *p* = < .001).

### a) Differences between persons living with dementia and persons without known cognitive impairments in relation to places visited outside home

The mean total number of reported places currently visited showed a significant difference (*p* < .001) between persons living with dementia and persons without known cognitive impairments. In contrast, the total number of applicable places were relatively similar and the difference was non-significant (*p* = .380), out of the 25 pre-listed types of places. The number of places that were affected by change (past or future) was significantly higher among the group of persons living with dementia (*p* < .001). The number of occupational gaps reported was also significantly higher (*p* < .001) (see Table 1).

More specifically, when looking at the results by places and domains (see Table 2), the group of persons living with dementia currently visited significantly fewer places than those in the comparison group in the domains A: commercial and administrative places (*p* < .001, specifically A1, A4, A6, and A7); C: social, cultural, and spiritual places (*p* = .002, specifically C6); and D: recreational and physical places (*p* = .010, specifically D4 and D7). In contrast, places in domain B: places for medical care were significantly more visited (*p =* .010, specifically B5) by the group of persons living with dementia.

### b) Differences between persons living with dementia and persons without known cognitive impairments in relation to maintenance and abandonment of places outside home

Table 2 goes into more detail showing that the group of persons living with dementia had abandoned more places than the comparison group. The hierarchy presented in Table 3 shows the type of places that were most abandoned (top) or most retained (bottom) for both groups. The “sports facility,” the “cottage or summer house,” and the “transportation centre” items illustrate a high abandonment ratio for both groups. In contrast, the “bank or post office” and the “entertainment or cultural places” had been abandoned to a much higher degree by the group of persons living with dementia than the comparison group (see Table 3.). The places above the line in Table 3 display in a visual descriptive way those most affected by change between past and present. Out of seven places that most of the group of persons living with dementia seemed to have retained, three were healthcare types of places. In addition to those, persons living with dementia were limited to the garden outside their house, their neighbourhood, going to visit their family or friends, and going to a restaurant or café. That group were experiencing a higher rate of change of visiting ratio (26.75%) than the comparison group (10.88%), (*p* < .001).

### c) Relationships between perceived occupational gaps and places outside home visited among persons living with dementia and persons without known cognitive impairments

Although there was a significant difference between the groups for the total number of occupational gaps (see Table 1), only seven occupations out of the 30 in OGQ demonstrated significant differences between the groups on item level. Two out of these seven items concern an activity performed outside the home (i.e., OGQ item 23: Involvement in activities in societies, clubs or unions (*p* = .013), and item 6: Doing heavy-duty maintenance of home, garden, car (*p* = .023).

Negative relationships between perceived occupational gaps and the total number of currently visited places (*r*_s_ = −.37 *n* = 70, *p* < .01) were found, which overall indicates that the more places visited, the lower the number of occupational gaps. There was however a difference between the correlation coefficients for the group of persons living with dementia (*r*_s_ = −.11, *n* = 35, *p* = .55), and the comparison group (*r*_s_ = −.46, *n* = 35, *p* < .01). So, the overall assumption that the more places visited, the lower perceived occupational gap was only empirically supported for the comparison group, but not for the group of persons living with dementia.

## Discussion

The results seem to indicate that participation in activities and places—as in going to places, visiting them, and performing activities there—was more affected both at home (OGQ) and outside home (ACT-OUT) for the group of persons living with dementia. Significant differences between groups could be seen, but they only apply to a limited number of items. While a shrinking of the outside world seems likely (Duggan et al., 2008), the way it would shrink might not be regular, identical for each place, nor straightforward. Rather, we surmise that it could be more nuanced, complex, and might be related to the disengagement observed in life space of older adults, also linked to driving cessation (Argyle et al., 2017; Marottoli et al., 2000). Our results seem to indicate a shift of participation in the group of persons living with dementia from places related to commercial, social, and recreational activities toward places related to self and medical care, with an increase in the B domain of medical care. These places are also usually reached by car, and persons not driving might be less inclined to ask for being driven to places related to leisure than places related to health (Pristavec, 2016). Although the questions in ACT-OUT do not address the meaning held by specific places, earlier research has underlined that there are places that are important, like for example the neighbourhood (Ward et al., 2017) or the grocery store (Brorsson et al., 2013). One of those specific places might very well be the day hospital for which a similar study also reported an increase of visits by persons living with dementia (Gaber, et al., 2019). There seems to be an increased use of medically oriented places in our sample, especially the day hospital, which would be expected in a society with a strong medical system like Switzerland.

There are commonalities in places abandoned or retained with a similar group comparison study in Sweden (Gaber et al., 2019). In both studies, the sports facility and transportation centre were among the most abandoned places for both groups. Day care and neighbourhood were among the most maintained places for the persons living with dementia in both studies; but for the comparison group, it was hairdresser, supermarket, and neighbourhood. There seems to be more diversity in places maintained for the comparison group, indicating that having dementia might influence the types of places one is more prone to visit. Although the local context in which older adults live influences the places they visit. This influence might be more visible for the comparison group than the group of persons living with dementia. Having a diagnosis of dementia would direct individuals to healthcare services in countries such as Sweden and Switzerland, which might explain the similarities seen in the results of the types of places abandoned or maintained. Also, a number of intrinsic and extrinsic factors, including coping strategies in using transportation, driving status, accessibility to public transportation (Womack et al., 2016), and societal and cultural organization, could cause the differences seen in both studies. Although there are variations seen in the specific order of abandonment between the two groups and between this study and Gaber et al.’s (2019) study, there are more places affected by change and to a higher degree for the persons living with dementia in both studies. Of course, not everyone diagnosed with dementia will find themselves limited to these places when going out, but in comparison to the group living without dementia, the ratio of abandonment for all other places is considerable, picturing a world that might be shrinking for most participants. Although a shrinking trend seems likely, to better understand what repercussions abandoning some types of places versus other types might have on participation, it would need to be further explored.

In considering the total number of places currently visited as an essential component of participation, there is an implicit assumption. Going to many and various spaces—that could be places for some individuals—would indicate an association between the number of places visited and the quality of perceived participation. It is important to distinguish between what the ACT-OUT is measuring (number and types of places visited, change between past and present) and how this measure is interpreted; and that not all items can be considered places for all participants. It might not be the quantity or diversity of items/places visited that indicates adequate, sufficient and satisfactory perceived participation for the individual, but rather the meaning given to the places by the occupations performed. It would have been interesting also to investigate whether places visited were experienced as valued, especially in regard to the pattern of abandonment and retention of specific places (Farias & Laliberte Rudman, 2016). Perceptions and experiences of out-of-home participation are constituted through doing occupations in particular places (Andrews et al., 2013). Out-of-home participation might not be apprehended solely by looking at places visited, but one would need to scrutinize the occupations that are being performed. Further research is needed to determine whether the perception of participation outside home is linked to the meanings given to places visited.

## Study Limitations

The chosen design of a cross-sectional study might be understood as a limit, as differences were determined through reported change between past, present, and future in ACT-OUT. Only differences between groups could be identified. Longitudinal designs would be needed in future studies to capture change—rather than differences—between past and present in participation outside home (Hedman et al., 2017).

Out-of-home participation being a complex, contextualized, and multifaceted concept, ACT-OUT only offers partial indicators, through identifying the places visited, and as recalled by participants. That said, it offers an insight into the patterns of spaces and/or places that constitute the outside world of individuals at present, compared to the past, and as envisioned in the future. It is a limitation to ask persons with memory problems to recall going to specific locations, so we supported it via using simple phrasing of questions and flexibility in approach in a face-to-face interview and allowing for any timeframe in the answers given. Also, using places visited as an indicator of perceived participation outside home might lead the reader to an understanding of participation as subjective and opposing it to objective components in a dichotomous vision. Perceived participation, using a transactional perspective (Cutchin & Dickie, 2013; Margot-Cattin, 2018), would not need to be considered as subjective (nor objective), but rather as experiences of occupations embedded in places. This perspective would then rely on self-report from persons living with dementia, rather than considering it as a study limitation, as there is a better recognition of their ability to share their experiences (Alzheimer Europe, 2011; Bethell et al., 2018). This study recognizes the importance for persons living with dementia to be involved in research activities, and values their perceptions of participation in places and activities, while recognizing the difficulty of measuring out-of-home participation (Bosco et al., 2019).

## Conclusion

This study suggests that older adults living with dementia report fewer visited out-of-home places than older adults living without dementia, although not in a clear, uniform, and straightforward way. There seems to be more of a shift from participation in places and activities for social, recreational, and commercial activities, toward places for health and medical care among persons living with dementia. However, it may not be only the number of places visited that indicates consequential participation, but there would be a need to also scrutinize occupations performed in those places.

### Key messages

- Persons living with dementia face a shrinking world with a shift from visiting recreational and cultural places toward more medically oriented places.
- It is important for occupational therapists to attend to their clients’ occupational gaps and participation in places outside home, in recognition of participation as a fundamental human right for persons living with dementia.
- Occupational therapists may support participation outside home not only in places that persons living with dementia seem to maintain (neighbourhood, day hospital, and restaurants), but also in places they might have abandoned, for example, nature places (parks and forests).

## Notes

1.
https://www.zas.admin.ch/zas/en/home/bases-legales-et-coordination-internationale/le-systeme-des-trois-piliers.html

2. Inspired and adapted from Gaber et al. (2019), with the permission of authors.
